# A Mini Review: The Potential Biomarkers for Non-invasive Diagnosis of Pulpal Inflammation

**DOI:** 10.3389/fdmed.2021.718445

**Published:** 2021-12-19

**Authors:** Brahmleen Kaur, Yoshifumi Kobayashi, Carla Cugini, Emi Shimizu

**Affiliations:** 1 Department of Oral and Maxillofacial Surgery, Rutgers School of Dental Medicine, Newark, NJ, United States,; 2 Department of Oral Biology, Rutgers School of Dental Medicine, Newark, NJ, United States,; 3 Department of Endodontics, Rutgers School of Dental Medicine, Newark, NJ, United States

**Keywords:** vital pulp therapy, pulp diagnosis, pulp inflammation, non-invasive method, biomarkers, gingival crevicular fluid

## Abstract

For assessing the adequacy of vital pulp therapy for an inflamed pulp, the use of non-invasive diagnostic tools is necessary to avoid further damage to the teeth. Detection of biomarkers that are indicative of the inflammatory status in pulp can be a promising tool for this purpose. These biomarkers need to be reliably correlated with pulpal inflammation and to be easily detected without pulp exposure. This mini-review article aims to review biomarkers that are present in gingival crevicular fluid (GCF) in inflamed pulp conditions. Several studies have reported the availability of various biomarkers including cytokines, proteases, elastase, neuropeptides, and growth factors. Non-invasive pulpal diagnostic methods will be useful as well to determine reversibility, irreversibility, or necrosis of inflamed pulp. These types of molecular diagnoses *via* analyzing the proteome have revolutionized the medical field, and are one of the most promising empirical methodologies that a clinician can utilize for the proactive identification of pulpal disease.

## INTRODUCTION

The decision to perform vital pulp therapy or to extirpate the pulp is based on the clinician’s diagnosis regarding the inflammatory status of the pulp caused by a carious lesion or traumatic injury. The modalities for assessing the pulpal condition are dependent on the patient’s chief complaint, the history of symptoms, clinical and radiographic assessment, pulpal sensibility, and periapical tests (1, 2). However, there is a dearth of high-quality studies claiming the insu cient accuracy of those modalities to determine the status of pulp inflammation (1). Additionally, there is no special test that measures the ability of the pulp to recover from inflammation. Thus, the diagnosis distinguishing reversible pulpitis from irreversible pulpitis lacks a strong foundation. Lastly, pulp diagnoses based on the current modalities have a poor correlation with the histopathological condition of the pulp (3–5).

As with any inflammatory process, molecular mediators are involved in the suppression and progression of the inflammatory response in the dental pulp. Gingival crevicular fluid (GCF) is easily collected, contains these mediators secreted from dental pulp *via* dentinal tubules (6). Therefore, the molecular mediators derived from the pulp in various stages of disease can be useful clinical tools to establish an accurate pulp diagnosis. The aim of this mini-review is to discuss the molecular mediators in inflamed dental pulp and evaluate the biomarkers present in GCF.

## PULPAL INFLAMMATION

Pulp inflammation is induced by a variety of causes: microbial intrusion via dental caries, cracks, or dentinal tubules; irritation by chemicals from etching and/or bonding materials for fillings; mechanical irritation during restorative procedures; or trauma caused by occlusion or orthodontic movement of the teeth (7). A series of inflammatory events are triggered to start the repair process (8). Pulp inflammation leads to changes in cellular composition and decrease cellular function, but in parallel, it facilitates an increase in mineral deposition from odontoblasts (9). The processes involved in the stages of inflammation occur both at cellular and molecular levels. The activation of the molecular response leads to the recruitment of immune cells (10). Dental pulp with reversible pulpitis shows coagulation and liquefaction in the tissue without bacteria, whereas dental pulp with irreversible pulpitis is characterized by the presence of acute inflammatory cells, mainly neutrophils, in the tissue underneath the lesion with bacterial infection (11–13). In irreversible pulpitis, lysosomal enzymes produced by neutrophils cause tissue damage and suppuration in dental pulp (14).

## METHODS FOR NON-INVASIVE PULP DIAGNOSIS

Currently, five non-invasive pulp diagnosis methods are broadly recognized: cold pulp test, heat pulp test, electric pulp test (EPT), laser Doppler flowmetry (LDF), and pulse oximetry (PO). These methods, especially the latter two, are highly accurate (84, 72, 82, 97, and 97%, respectively) in testing pulp vitality (15). Importantly, the main purpose of these tests is to diagnose pulp vitality, not necessarily to distinguish reversible from irreversible pulpitis. The standard diagnostic procedure to distinguish between the two types of pulpitis is the cold pulp test. However, the response to thermal stimulus is a ected by age of the patient. Individuals >53 years old have an increased incidence of painless pulpitis (non-lingering pain) than the ≤33 year old group (16), indicating that thermal test is not absolutely precise for pulp diagnosis.

## ROLE OF BIOMARKERS IN ORAL DISEASES

The role of biomarkers for the diagnosis of oral cancers, temporomandibular joint disorders (TMD), periodontal disease, and caries have been actively investigated (17–19). Tumor necrosis factor (TNF) and TNF receptor (TNFR) 2 have been identified as biomarkers for diagnosis as well as treatment in terms of reduction of inflammation and pain of TMD (20). Salivary microRNAs have been studied as possible diagnostic and prognostic biomarkers for oral cancers and periodontal disease (18). Proteomic studies using mass spectrometry have been performed in order to correlate proteomes with periodontal disease conditions (21). Gingival crevicular fluids (GCF), saliva, and blood serum are mainly tested in those studies (22). Other promising biomarkers involved in periodontal inflammatory pathology are interleukin (IL)-1β, matrix metalloproteinase (MMP)-8, and serum carboxy-terminal telopeptide of type I collagen (ICTP) (23). In early childhood, a significant correlation has been established between the presence of cavities and the high concentrations of protective proteins in saliva, including IgA, IgG, immunoglobulins, β-defensin-2, and histatin-5 (24, 25), which have the potential to be utilized as caries biomarkers (19). Several genetic alterations and proteomic changes have been evaluated to identify the specific indicators of risk for development of oral cancers (26).

## BIOMARKERS ASSOCIATED WITH PULPAL INFLAMMATION

This mini review provides an overview of molecules that are present and measurable during pulpal inflammation and therefore have the potential to serve as biomarkers for irreversible pulpitis by non-invasive detection methods such as GCF sampling. The pulp cavity communicates with the periodontal ligament *via* the apical foramina, accessory lateral and furcation canals, and dentinal tubules. Therefore, the molecular mediators of inflammation are present not only in pulp tissue and the blood, but also in GCF and dentinal fluid (14, 27, 28).

The potential biomarkers for the diagnosis of irreversible pulpitis are listed in Table 1. The selected biomarkers have shown statistical significance in previous literatures, when measuring cellular inflammation by the quantitative methods, such as enzyme-linked immunosorbent assay (ELISA), western blot analysis, quantitative reverse transcription PCR (RT-qPCR), radioimmunoassay, multiple assay, and enzyme assay.

## THE SELECTED PULPITIS BIOMARKERS WHICH ARE DETECTED IN GCF

The biomarkers described below are detected in GCF and are upregulated reflecting pulp inflammation. Since these biomarkers are upregulated also in the cases of periodontitis, measuring solely these markers may not distinguish pulp inflammation and periodontitis. There is no perfect solution to this problem, however, the detection of periodontitis markers might negatively select pulpitis patients. For example, cross-linked N-terminal telopeptides of type I collagen (NTx), a bone resorption marker, is reported to be detected only in the cases of periodontitis, but not in gingivitis cases (70). As bone resorption specifically occurs with periodontitis or orthodontic treatment, NTx should not be detected from pulpits patients without periodontitis. Likewise, the detection of the other bone resorption markers, such as Cathepsin-K, RANKL, or IL-34, can aid distinguishing periodontitis from pulpitis. This type of strategies to avoid “bias” by the biomarkers of periodontitis will be discussed later, as distinguishing pulpitis from other inflammatory conditions will be a central challenge to utilize biomarkers in GCF for non-invasive pulp diagnosis.

### Matrix Metalloproteinase-8

MMP-8 is mainly produced by neutrophils, and it degrades type I, II and III collagens. It has a significant role in the regulation of the innate immune system, especially by activating IL-6 and IL-8 (71). MMP-8 is the most abundant collagenase in dentine (72). MMP-8 is detected in inflamed pulp and periapical root-canal exudates (73). Higher MMP-8 is included in dentin samples from reversible or irreversible pulpitis patients (52). In pulpitis patients, MMP-8 levels were significantly higher in the GCF samples compared with the healthy group (35). Patients’ subjective pain levels were significantly related to both MMP-8 and substance P (SP) levels. MMP-8 and SP levels in GCF were decreased during root canal treatment, and they showed a positive correlation with each other (74). As described above, MMP-8 is one of the hopeful biomarkers for pulp inflammation diagnosis, however, MMP-8 is also detected in GCF from chronic periodontitis patients (75, 76).

### Chemokine (C-X-C Motif) Ligand 8/Interleukin-8 (CXCL8/IL-8)

IL-8 is a chemoattractant cytokine produced and released by many different cell types in inflamed pulp tissues, such as monocytes/macrophages, lymphocytes, fibroblasts, endothelial cells, and odontoblasts (29). It recruits neutrophils and facilitate their accumulation at the injured site (77, 78). Levels of IL-8, as well as ratio of IL-6/IL-8 and IL-8/IL-10, have been shown to be higher in irreversible pulpitis and in caries-exposed pulp as compared to normal pulp (34). These levels can be assessed in the blood from caries-exposed teeth to evaluate if vital pulp therapy can be applied (34). In addition, Karapanou et al. and Akbal Dincer et al. have shown that IL-8 levels significantly increase in gingival crevicular fluid (GCF) of inflamed pulp teeth with a disease, such as irreversible pulpitis (33, 35). Since IL-8 is significantly increased in dentinal fluid of reversible and irreversible pulpitis compared to normal pulp, it can be useful for diagnosis of pulpitis (28). Therefore, IL-8 is a potential biomarker for pulp diagnosis using non-invasive method such as collection of GCF. However, IL-8 level is also enhanced in GCF of chronic periodontitis (79).

### Substance P

SP and NKA (neurokinin A, described below) are the members of tachykinin family, which are involved in neuronal excitation, vasodilatation, plasma extravasation, nociception, and proinflammatory actions on immune and inflammatory cells (80). SP is a neuropeptide that is secreted from sensory nerve endings and inflammatory cells such as neutrophils, monocytes/macrophages, eosinophils, lymphocytes, and dendritic cells (81). SP stimulates macrophage’s function by their NF-kB activation, which causes the increase of inflammatory chemokine secretion from macrophages (82). Several studies have shown by ELISA and radioimmunoassay that SP is significantly increased in inflamed pulp tissues (44–47). In addition, Akbal Dincer et al. have shown that SP is significantly higher in GCF from teeth with symptomatic irreversible pulpitis (SIP) in comparison with the healthy control group (35). However, SP and NKA are detected in GCF from periodontal disease patients with gingivitis or periodontitis (83).

### Neurokinin A

NKA binds to NK1R, initiating an inflammatory process through the NF-kB signaling in macrophage/monocyte cells; whereas the neurological functions of NKA are primarily mediated by NK2R (84). Heidari et al. and Akbal Dincer et al. have shown that NKA is significantly enhanced in GCF of teeth with SIP in comparison with asymptomatic teeth (35, 48). Although SP and NKA are also upregulated in GCF of teeth with periodontitis, the levels are reduced after periodontal treatment (85).

## THE SELECTED PULPITIS BIOMARKERS WHICH ARE DETECTED IN DENTINAL FLUID

The biomarkers listed below can be detected in dentinal fluid and they increase in the cases of pulp inflammation. The sampling of dentinal fluid requires the removal of enamel or soft dentine on caries; thus, it is not a non-invasive procedure presently. However, dentinal fluid can be collected without making access to patients’ pulp and contains cues from the odontoblasts contiguous to inflammatory site (28). It would be supportive to list the biomarkers below, expecting the development of new methodology to collect them non-invasively in future.

### Matrix Metalloproteinase-9

MMP-9 is a member of the MMPs, a family of zinc-dependent endopeptidases that play major roles in the degradation of extracellular matrix (ECM) during physiological and pathophysiological processes, including tissue remodeling (86). In inflammatory conditions, MMP-9 is locally produced and activated by infiltrating neutrophils (87, 88). MMP-9 is barely detectable in the healthy dental pulp, however, it is strongly increased in the dentinal fluid of inflamed pulp tissues with a disease, such as irreversible pulpitis (27, 28). However, since MMP9 is increased in dentinal fluid of both reversible and irreversible pulpitis (28), these two types of pulpitis cannot be distinguished by detecting MMP9 alone at this time.

### Interleukin-1α

Interleukin 1 alpha is a cytokine produced mainly by activated macrophages, as well as neutrophils, epithelial cells, and endothelial cells. IL-1α is involved in a variety of immune reaction, including the stimulation of thymocyte proliferation by inducing IL-2 release, B-cell maturation and proliferation, and fibroblast growth factor activity. IL-1α is involved in the inflammatory response (32, 89). IL-1α is increased in the dentinal fluid of irreversible pulpitis compared to normal pulp, however, there is not significant difference compared to reversible pulpitis (28).

### Interleukin-1β

IL-1β is a proinflammatory cytokine produced by activated macrophages. It induces neutrophil influx and activation, T-cell activation and cytokine production, B-cell activation and antibody production. It also plays a role in angiogenesis, working synergistically with TNF and IL-6 (90). IL-1β was found to increase in inflamed pulp tissue in analyses using ELISA (30), and RT-qPCR (39). IL-1β is increased in the dentinal fluid of irreversible pulpitis compared to normal pulp, however, it is not significantly different to reversible pulpitis (28).

### Interleukin-6

IL-6 is a cytokine synthesized mainly by macrophages and monocytes at sites of inflammation, which contributes to host defense in the acute phase immune response. IL-6 induces acute phase proteins such as C-reactive protein and serum amyloid A (91). It also regulates acquired immune response such as T cell development (92). Although several articles have shown IL-6 is upregulated in the tissues of irreversible pulpitis (32, 43), upregulation of IL-6 level in GCF in irreversible pulpitis has not been demonstrated. IL-6 is upregulated in dentinal fluid of pulpitis, however, there is not significantly different between reversible and irreversible pulpitis (28).

### Tissue Inhibitor Matrix Metalloproteinase 1

TIMP1 is a tissue inhibitor of metalloproteinase, playing a crucial role in ECM composition and wound healing (93). It also functions as a growth factor that regulates cell differentiation, migration, and apoptosis. After periapical lesion induction, the TIMP-1 mRNAs are expressed coordinately with mRNA of MMP-1, IL-6, and cyclooxygenase-2 (COX-2) (94). TIMP-1 is strongly detected in the dentinal tubuli of carious teeth, in contrast to sound teeth (95). TIMP1 is increased in the dentinal fluid of irreversible pulpitis compared to normal pulp, however, the intensity is not significantly different to reversible pulpitis (28).

### Tumor Necrosis Factor Alpha

TNFα is a proinflammatory cytokine produced by a variety of cell types, such as macrophages, T lymphocytes, and natural killer cells during inflammation (96). It causes a disruption of the macrovascular and microvascular circulation, and also, stimulates apoptosis and acute inflammation (97, 98). Several studies have shown that TNFα is highly expressed in inflamed dental pulp tissue (36–38). In addition, TNFα is increased in the dentinal fluid of reversible and irreversible pulpitis (28).

### Fibroblast Growth Factor 1

Fibroblast growth factor-1 plays a vital role in cell survival, cell division, angiogenesis, cell differentiation migration. It was reported to be involved in odontoblast differentiation together with TGFβ (99). FGF1 significantly increased in the dentinal fluid of irreversible pulpitis compared to reversible pulpitis or normal pulp (28). When FGF1, IL-1α, IL-6, and TIMP-1 biomarkers are combined, significant improvement of discrimination in the detection of IP versus RP diagnosis was shown (28).

### Vascular Endothelial Growth Factor A

Vascular endothelial growth factor A induces angiogenesis, vasculogenesis, and endothelial cell growth, primarily through its interactions with the VEGFR1 and -R2 receptors on endothelial cells. It enhances the chemotaxis of cells to the inflamed site (89). It is an essential component also for dental pulp repair in response to damage (100). VEGF-A/VEGFR2 axis promoted the migration of hDPSCs *via* the FAK/PI3K/Akt and p38 MAPK signaling pathways (101). With a multiplex assay of dentinal fluid, Brizuela and colleague found that the concentration of VEGF-A is significantly higher in the fluid from teeth with SIP, compared to that from normal teeth or theeth with reversible pulpitis (28). This finding indicated the possibility of distinguishing irreversible pulpitis and reversible pulpitis.

## PROSPECTS FOR NON-INVASIVE PULP DIAGNOSIS USING BIOMARKERS

Currently, GCF is the single source for biomarkers to non-invasively diagnose pulpitis. Since the contents of GCF reflect the condition of surrounding tissues including gingiva, periodontal ligament (PDL) and alveolar bone (102), periodontitis is a major cause of inflammatory biomarker contamination into the GCF (103). Several approaches to eliminate this ‘bias’ have been proposed (14). In cases where the patients have periodontal inflammation, clinicians could: (i) average the values taken from several sites on one or multiple teeth, (ii) combine biomarker data with radiographic observations, or (iii) define a specific pattern of metabolites relevant to the pulp and not the periodontium. The third approach is theoretical at present because no such metabolites have been detected in the GCF, however, there is a set of reports predicting the feasibility of such a method. In teeth with caries, pulp cells were found to upregulate the expression of *CARD18/ICEBERG*, a caspase recruitment domain family member 18 gene, more than 10-fold that of normal pulp (104). In contrast, a group of genes regulating the inflammasome, including *CARD18/ICEBERG*, is downregulated in the gingival tissue cells from periodontal disease patients (105). These observations show an example of differential gene expression patterns between dental pulp and gingival tissue in inflammatory conditions. Although these gene products have not been detected in GCF, the development of protein-detection technology, and obviously, comprehensive gene profiling of pulp and gingival tissue in inflammatory condition, would make it possible to identify pulpitis-specific proteins in GCF.

## CONCLUSION

In this mini review, we outlined the potential candidates for biomarkers in the context of pulpal diagnostics by non-invasive sampling methods. The most promising candidates are MMP-8, IL-8, Substance P, and Neurokinin A, owing to their presence in GCF and upregulation in SIP patients. The importance of identifying such biomarkers is obvious given the increasing need for vital pulp therapy, however, those candidates have also been reported to increase in GCF from periodontitis patient. In the future, comprehensive gene profiling of pulp and gingival tissue in inflammatory conditions and the development of protein detection technologies may aid in the identification of pulpitis-specific proteins in the GCF.

## Figures and Tables

**TABLE 1 |:** The potential biomarkers for diagnosis of irreversible pulpitis.

Biomarkers (gene/protein symbols)	Function	References	Analysis method	Collected sample	Results NP vs. IP (*p*-value)
Chemokine (C-X-C motif) ligand 8/Interleukin-8 (CXCL8/IL8)	A chemoattractant cytokine produced and released by many different cell types in inflamed tissues.	(29)	ELISA	DP	0.08 vs. 1.82 pg/ml (*p* = 0.037)
		(30)	ELISA	DP	2.05 vs. 45.77 pg/ml (*p* < 0.001)
		(31)	Multiple assay	DP	0.23 vs. 371.4 pg/mg (*p* < 0.01)
		(32)	RT-PCR	DP	1,200 times upregulation in IP (*p* < 0.005)
		(33)	Cytokine assay	GCF	173.8 vs. 302.1 pg/ml (*p* = 0.0231)
		(34)	ELISA	Blood	10.06 (CE) vs. 42.08 pg/mg (*p* < 0.05)
		(6)	Microarray	DP	36.96 times upregulation (*p* < 0.001)
		(35)	ELISA	GCF	Upregulation in IP (*p* < 0.001)
		(28)	Multiple assay	DF	0.13 vs. 1.125 pg/ml (*p* < 0.05)
Tumor necrosis factor alpha (TNFA/TNFα)	A proinflammatory cytokine produced by a variety of cell types, such as macrophages, T lymphocytes, and natural killer cells during inflammation.	(36)	ELISA	DP	64.01 vs. 1962.99 pg/g (*p* < 0.001)
		(31)	Multiple assay	DP	0 vs. 3.08 pg/mg (*p* < 0.01)
		(37)	RT-PCR	DP	0.591 vs. 1.119 Upregulation in IP (*p* = 0.002)
		(38)	RT-PCR	DP	Upregulation in IP (*p* =0.05)
		(39)	RT-PCR	DP	Upregulation in IP (*p* = 0.002)
		(34)	ELISA	Blood	0.099 vs. 1.929 pg/mg (*p* < 0.05)
		(28)	Multiple assay	DF	0.93 vs. 1.07 pg/ml (*p* < 0.05)
Matrix metalloproteinase-9 (MMP9)	A family of zinc-dependent endopeptidases that play major roles in the degradation of extracellular matrix.	(40)	ELISA	DP	Upregulation in IP (*p* < 0.001)
		(27)	MMP9 assay	DF	Upregulation in IP (*p* < 0.05)
		(41)	RT-PCR	DP	Upregulation in IP (*p* < 0.05)
		(42)	Western blot	DP	Upregulation in IP (*p* < 0.05)
		(6)	Microarray	DP	7.37 times upregulation (*p* < 0.001)
		(28)	Multiple assay	DF	50.5 vs. 1,271 pg/ml (p < 0.05)
Interleukin-6 (IL6)	A cytokine synthesized mainly by macrophages and monocytes at sites of inflammation, which contributes to host defense in the acute phase immune response.	(43)	ELISA	DP	0.01 vs. 36 pg/mg (*p* < 0.0001)
		(31)	Multiple assay	DP	0 vs. 7.82 pg/mg (*p* < 0.05)
		(32)	RT-PCR	DP	282 times upregulation in IP (*p* < 0.02)
		(34)	ELISA	Blood	0.024 (CE) vs. 0.159 pg/mg (*p* < 0.05)
		(6)	Microarray	DP	2.3 times upregulation (*p* < 0.001)
		(28)	Multiple assay	DF	0.56 vs. 0.62 pg/ml (*p* < 0.05)
Substance P (SP)	A neuropeptide secreted from sensory nerve endings and inflammatory cells such as neutrophils, monocytes/macrophages, eosinophils, lymphocytes, and dendritic cells.	(44)	RIA	DP	2.0 vs. 3.6 ng/g (*p* = 0.02)
		(45)	RD, RIA	DP	18.2 vs. 147.7 pM (*p* = 0.001)
		(46)	RIA	DP	0.33 vs. 154.37 pg/mg (*p* < 0.05)
		(47)	ELISA	DP	36 vs. 0.01 pg/mg (*p* < 0.05)
		(35)	ELISA	GCF	Upregulation in SIP (*p* < 0.005)
Interleukin-1 beta (IL1B)	A member of the IL-1 family cytokines produced by activated macrophages, mediating the inflammatory response, involved in cell proliferation, differentiation, and apoptosis.	(30)	ELISA	DP	3.72 vs. 28.02 pg/ml (*p* < 0.001)
		(31)	Multiple assay	DP	2.11 vs. 12.34 pg/mg (*p* < 0.01)
		(39)	RT-PCR	DP	Upregulation in IP (*p* < 0.001)
		(28)	Multiple assay	DF	0.34 vs. 1.23 pg/ml (*p* < 0.05)
Neurokinin A (NKA)	A members of tachykinin family, which binds to NK1R, which initiates an inflammatory process through the NFkB signaling in macrophage/monocyte cells.	(44)	RIA	DP	2.0 vs. 8.5 ng/g (*p* < 0.001)
		(46)	RIA	DP	73.33 vs. 194.98 pg/mg (*p* < 0.05)
		(48)	ELISA	GCF	1.84 vs. 2.23 pg/ml (p < 0.05)
		(35)	ELISA	GCF	Upregulation in IP (*p* < 0.001)
Calcitonin gene-related peptide (CGRP)	A potent peptide vasodilator produced in both peripheral and central neurons.	(44)	RIA	DP	27.1 vs. 49.8 ng/g (*p* = 0.03)
		(49)	Flow cytometry	DP	Higher percentages of CGRP expression in IP 5.018 vs. 0.308% (*p* < 0.005)
		(46)	RIA	DP	212.71 vs. 619.41 pg/mg (*p* < 0.05)
Matrix metalloproteinase-2 (MMP2)	Degrades type IV collagen and regulates vascularization and the inflammatory response.	(40)	ELISA	DP	Downregulation in IP (*p* < 0.001)
		(50)	ELISA	DP	22.2 vs. 81.4 ng/mg (*p* < 0.05)
		(51)	Zymography	DP	16,308 vs. 28,465 arbitrary units (*p* < 0.05)
Matrix metalloproteinase-3 (MMP3)	Degrades type-II, III, IV, IX, and X collagen, proteoglycans, fibronectin, laminin, and elastin and participates in wound repair.	(40)	ELISA	DP	Downregulation in IP (*p* < 0.001)
		(50)	ELISA	DP	108.6 vs. 1,072 ng/mg (*p* < 0.05)
Matrix metalloproteinase-8 (MMP8)	A member of the matrix metalloproteinase (MMP) family released mainly from neutrophils, which stimulates anti-inflammatory function of macrophages.	(35)	ELISA	GCF	Upregulation in IP (*p* < 0.001)
		(52)	ELISA	Dentin	0.0691 vs. 1.975 ng/ml (RP: 0.2132) (*p* < 0.001)
Interleukin-1 alpha (IL1A)	A cytokine produced by activated macrophages, which stimulates thymocyte proliferation. Involved in the inflammatory response.	(31)	Multiple assay	DP	6.04 vs. 31.13 pg/mg (*p* < 0.001)
		(28)	Multiple assay	DF	11.15 vs. 15.21 pg/ml (*p* < 0.05)
Interleukin-4 (IL4)	A key regulator in humoral and adaptive immunity. Activates M2 macrophages alleviating pathological inflammation.	(53)	ELISA	DP	0.63 vs. 2.46 ng/ml (*p* < 0.001)
		(31)	Multiple assay	DP	0.9 vs. 3.18 pg/mg (*p* < 0.05)
Prostaglandin E2 (PGE2)	Acts as a component in feeling pain *via* inflammatory nociception and regulates various lymphocyte functions.	(54)	RIA	DP	Upregulation in IP (*p* < 0.01)
		(55)	ELISA	DP	0.57 vs. 152.06 ng/ml (*p* < 0.01)
Chemokine (C-C motif) ligand 2 (CCL2/MCP1)	Strong chemotactic activity for monocytes and basophils by binding to the receptor CCR2.	(31)	Multiple assay	DP	16.83 vs. 110.7 pg/mg (*p* < 0.05)
		(6)	Microarray	DP	3.61 times upregulation (*p* < 0.001)
Catalase (CAT)	Protects cells from hydrogen peroxide and promotes growth of various types of cells.	(56)	Enzyme assay	DP	1,126 vs. 3,074 mU/ml (*p* < 0.01)
		(57)	Enzyme assay	DP	1.61 vs. 2.44 mU/mg (2.99 in RP) (*p* < 0.05)
Cyclic GMP Phosphodiesterase (cGMP PDE)	Hydrolysis of cAMP or cGMP.	(58)	Enzyme assay	DP	4.5 times upregulation (*p* < 0.05)
Cyclic AMP Phosphodiesterase (cAMP PDE)		(59)	Enzyme assay	DP	4.5 times upregulation (*p* < 0.05)
Bradykinin	Induces vasodilatation and exerts an anti-inflammatory effect.	(60)	RD, RIA	DP	19.41 vs. 262.26 fmol/ml (*p* < 0.05)
Chemokine (C-C motif) ligand 11/Eotaxin (CCL11)	A cytokine belonging to the CC chemokine family, which recruits eosinophils into sites of inflammation.	(31)	Multiple assay	DP	0 vs. 11.22 pg/mg (*p* < 0.01)
Chemokine (C-C motif) ligand 22 (CCL22/MDC)	A chemokine produced by macrophages, dendritic cells, B cells, and T cells, controlling pathogenesis of allergy, and suppresses adaptive immune response.	(31)	Multiple assay	DP	17.41 vs. 56.79 pg/mg (*p* < 0.05)
Chemokine (C-C motif) ligand 7 (CCL7/MCP3)	An inflammatory cytokine produced by macrophages, which stimulates the recruitment of monocytes and neutrophils to injured site.	(31)	Multiple assay	DP	1.93 vs. 13.1 pg/mg (*p* < 0.05)
chemokine (C-X-C motif) ligand 1 (CXCL1/GRO)	A chemoattractant for neutrophils to the site of injury or infection.	(31)	Multiple assay	DP	2.58 vs. 53.09 pg/mg (*p* < 0.05)
Cluster of Differentiation 86 (CD86)	A critical costimulatory molecule in T cell activation and proliferation by binding to CD28 antigen on T cells.	(6)	Microarray	DP	4.53 times upregulation (*p* < 0.001)
Copper-zinc superoxide dismutase (SOD1)	An antioxidant enzyme protecting the cell from reactive oxygen species toxicity, suppressing pro-inflammatory immune responses.	(61)	RT-PCR	DP	0.91 vs. 5.16 Upregulation in IP (*p* < 0.05)
C-X3-C Motif Chemokine Ligand 1/Fractalkine (CXCL1)	A member of the CXC chemokine family, which acts as a chemoattractant for neutrophils at injured site.	(31)	Multiple assay	DP	146.8 vs. 14.82 pg/mg (*p* < 0.05)
C-X-C Motif Chemokine Ligand 10 (CXCL10)	An inflammatory chemokine of the CXC chemokine family, which recruits NK cell at injured site.	(62)	RT-PCR	DP	Upregulation in IP (*p* < 0.05)
C-X-C Motif Chemokine Ligand 12 (CXCL12)	A strong chemoattractant for T/B-lymphocytes, inflammatory monocytes and mediator of pathogenic inflammation.	(63)	RT-PCR	DP	31.87 vs. 311.62, 10 times upregulation (*p* < 0.0001)
Defensin Beta 1 (DEFB1)	A peptide produced mainly by epithelial cells, which acts as an antimicrobial function.	(39)	RT-PCR	DP	Upregulation in IP (*p* = 0.005)
Defensin Beta 4 (DEFB4)	Presents antimicrobial activity against Gram-negative bacteria and Gram-positive bacteria.	(39)	RT-PCR	DP	Upregulation in IP (*p* = 0.033)
Elastase (ELANE)	Breaks down elastin. An enzyme secreted from neutrophils, which controls the pathologic processes of a variety of inflammatory diseases.	(55)	ELISA	DP	17.14 vs. 123.37 μg/ml (*p* < 0.05)
Endothelial nitric oxide synthase (NOS3)	An enzyme mainly expressed in the endothelial cells. Controls nitric oxide (NO) which is related to vascular smooth muscle relaxation.	(64)	RT-PCR	DP	Upregulation in IP (*p* < 0.05)
Ephrin type-A receptor 7 (EphA7)	One of the ephrin receptor subfamily (the protein-tyrosine kinase family), which controls cell growth and apoptosis.	(65)	RT-PCR	DP	59.5 vs. 180 Upregulation in IP (*p* < 0.01)
Fibroblast Growth Factor Acidic (FGFA)	A member of FGF superfamily and mediator of vascular pathophysiology.	(28)	Multiple assay	DF	10.91 vs. 12.76 pg/ml (*p* < 0.05)
Fms-related tyrosine kinase 3 ligand (FLT3LG)	Acts as a growth factor to increase the numbers of immune cells.	(31)	Multiple assay	DP	103.9 vs. 8.39 pg/mg (*p* < 0.001)
Granulocyte colony-stimulating factor (G-CSF)	A major cytokine produced by fibroblasts, endothelial cells, and bone marrow stromal cells, stimulating proliferation, differentiation, and survival of neutrophil progenitors.	(31)	Multiple assay	DP	6.75 vs. 0.99 pg/mg (*p* < 0.05)
Granulocyte-macrophage colony-stimulating factor (GM-CSF)	An inflammatory cytokine produced by macrophages, T cells, mast cells, natural killer cells, endothelial cells, and fibroblasts. Stimulates differentiation and proliferation of granulocytes and macrophages derived from hematopoietic progenitor cells.	(31)	Multiple assay	DP	0.21 vs. 21.12 pg/mg (*p* < 0.001)
Histamine	An organic nitrogenous compound produced by mast cells and basophils, which induces vasodilation.	(53)	ELISA	DP	2.125 vs. 30.835 ng/ml (*p* < 0.001)
Interleukin-12 (IL12)	Involved in the differentiation of naive T cells into Th1 cells, activating natural killer cells, and having anti-angiogenic activity.	(31)	Multiple assay	DP	0 vs. 32.96 pg/mg (*p* < 0.01)
Immunoglobulin A (IgA)	A principal antibody class in the secretions and functions in immunity.	(55)	ELISA	DP	0.18 vs. 0.33 mg/ml (*p* < 0.05)
Immunoglobulin E (IgE)	Activates mast cell/basophil’s function. Plays a central role in acute allergic reactions and chronic inflammatory allergic diseases	(53)	ELISA	DP	0.21 vs. 0.81 IU/ml (*p* < 0.001)
Immunoglobulin G (IgG)	A major class of immunoglobulins, which protects against bacterial and viral infections.	(55)	ELISA	DP	1.82 vs. 3.81 mg/ml (*p* < 0.01)
Immunoglobulin M (IgM)	Acts as the first line of host defense against infections.	(55)	ELISA	DP	0.05 vs. 0.13 mg/ml (*p* < 0.01)
Inducible nitric oxide synthase (NOS2)	An enzyme produced by numerous cell types, which contributes to prolonged inflammation.	(64)	RT-PCR	DP	Upregulation in IP (*p* < 0.05)
Intercellular Adhesion Molecule 1 (ICAM1)	A transmembrane protein expressed in the vascular endothelium, macrophages, and lymphocytes, controlling leukocyte recruitment.	(6)	Microarray	DP	3.2 times upregulation (*p* < 0.001)
Interferon Alpha (IFNA)	An immunomodulatory and cytokine, which controls the development of autoimmunity.	(31)	Multiple assay	DP	38.88 vs. 18.52 pg/mg (*p* < 0.05)
Interferon gamma (IFNG)	A proinflammatory cytokine secreted by T-cells and NK cells, which controls inflammation and autoimmune disease.	(34)	ELISA	Blood	0 vs. 0.028 pg/mg (*p* < 0.05)
Interleukin-10 (IL10)	A cytokine produced mainly by monocytes, which prevent tissue damage to maintain normal tissue homeostasis.	(34)	ELISA	Blood	0.266 vs. 0.967 pg/mg (*p* < 0.05)
Interleukin-13 (IL13)	An immunoregulatory cytokine produced by allergic inflammation in many tissues.	(31)	Multiple assay	DP	2.6 vs. 0 pg/mg (*p* < 0.05)
Interleukin-18 (IL18)	A cytokine produced mainly by antigen-presenting cells, which modulates both innate and adaptive immune system.	(32)	RT-PCR	DP	10 times upregulation in IP (*p* < 0.005)
Interleukin-2 (IL2)	A cytokine produced by various immune system cells, which modulates immune responses and inflammation.	(66)	ELISA	DP	541.29 vs. 1944.75 Units/mg (*p* < 0.05)
Interleukin-7 (IL7)	A cytokine secreted by stromal cells, keratinocytes, dendritic cells, and epithelial cells, stimulating stem cell differentiation and modulates B and T cell development.	(31)	Multiple assay	DP	4.23 vs. 0 pg/mg (*p* < 0.001)
Lymphocyte Cytosolic Protein 2 (LCP2)	An adapter protein highly expressed in spleen, thymus, and peripheral blood leukocytes, which regulates normal T-cell development	(6)	Microarray	DP	2 times upregulation (*p* < 0.001)
Macrophage inflammatory protein-1 alpha (MIP1A)	A chemotactic chemokine secreted by macrophages, which recruits inflammatory cells and inhibition of stem cell activity.	(31)	Multiple assay	DP	6.23 vs. 52.02 pg/mg (*p* < 0.01)
Macrophage inflammatory protein-1 beta (CCL4/MIP1β)	A chemokine produced by monocytes, lymphocytes, neutrophils, fibroblasts, and endothelial cells, causing inflammation and fibrosis.	(31)	Multiple assay	DP	1.85 vs. 81.36 pg/mg (*p* < 0.001)
Manganese superoxide dismutase (SOD2)	Removal of mitochondrial reactive oxygen species (ROS) to protection of cell death.	(61)	RT-PCR	DP	0.85 vs. 15.42 Upregulation in IP (*p* < 0.05)
Matrix metalloproteinase-1 (MMP1)	Produced by fibroblasts, keratinocyte, endothelial cells, and monocytes/macrophages.	(50)	ELISA	DP	120.4 vs. 713.2 ng/mg (*p* < 0.05)
Myeloperoxidase protein (MPO)	A hemoprotein produced from neutrophils and macrophages, which catalyzes the conversion of chloride and hydrogen peroxide to hypochlorite.	(51)	MPO assay	DP	Upregulation in IP (*p* < 0.05)
Neurokinin 1 receptor (NK1R)	A mediator of pain sensitivity and neurogenic inflammation, which functions in the nervous system.	(67)	Radioreceptor assay	DP	7.4 vs. 15.83 pmol/100 μg (*p* < 0.01)
Neuropeptide Y (NPY)	A potent modulator of immune responses produced by T cells, macrophages, and dendritic cells during inflammation.	(46)	RIA	DP	160.39 vs. 588.63 pg/mg (*p* < 0.05)
Nucleotide-binding oligomerization domain-containing protein 2 (NOD2)	A cytosolic receptor belonging to NOD-like receptor (NLR) family, which localizes in inflamed areas of damaged tissues.	(38)	RT-PCR	DP	Upregulation in IP (*p* = 0.05)
Osteocalcin (BGLAP)	A glycoprotein presented in bone and dentin matrix, which is involved in chronic inflammation.	(31)	Multiple assay	DP	Upregulation in IP and RP. RP is higher than IP (*p* < 0.05)
Receptor for advanced glycation end products (RAGE)	A multiligand receptor expressed in endothelial cells, myeloid cells and lymphocytes, which is related to chronic inflammation.	(68)	RT-PCR	DP	0.12 vs. 2.13 Upregulation in IP (*p* < 0.001)
Receptor-type tyrosine-protein phosphatase C (PTPRC)	A signaling molecule expressed in nucleated cells of hematopoietic origin, playing a critical role in immune cell function, including B and T cell receptor signaling.	(6)	Microarray	DP	3.14 times upregulation (*p* < 0.001)
Sodium channel protein type 10 subunit alpha (SCN10A/Nav1.8)	A sodium ion channel subtype expressed in adult sensory neurons, which is related to inflammatory pain.	(42)	Western blot	DP	Upregulation in IP (*p* < 0.05)
Sodium channel protein type 11 subunit alpha (SCN11A/Nav1.9)	A voltage-gated sodium channel expressed in nociceptive neurons of dorsal root ganglia and trigeminal ganglia, acting as a major effector of peripheral inflammatory pain hypersensitivity.	(42)	Western blot	DP	Upregulation in IP (*p* < 0.05)
Spi-1 Proto-Oncogene (SPI1)	A member of the E26-transformation-specific family expressed in leukocytes and fibroblasts, which controls the self-renewal of hematopoietic stem cells.	(6)	Microarray	DP	2.06 times upregulation (*p* < 0.001)
Tissue Inhibitor of metalloproteinase-1 (TIMP1)	Regulates matrix metalloproteinases (MMPs), remodeling of extracellular matrix formation and inflammatory activity.	(28)	Multiple assay	DF	6.07 vs. 20.57 pg/ml (*p* < 0.05)
Tissue Inhibitor of metalloproteinase-2 (TIMP2)	A strong inhibitor of MT1-MMP.	(51)	ELISA	DP	38.89 vs. 244.8 ng/ml (*p* < 0.05)
Tissue-type plasminogen activator (tPA)	A major intravascular activator of fibrinolysis and secreted by vascular endothelial cells.	(69)	RT-PCR	DP	Upregulation in IP (*p* = 0.025)
Toll-like receptor 8 (TLR8)	Recognizes the pathogens and activates innate immunity by increasing production of pro inflammatory mediators.	(6)	Microarray	DP	2.33 times upregulation (*p* < 0.001)
Transforming growth factor alpha (TGFA/TGFα)	A mitogenic polypeptide produced by immune cells, which regulates wound healing.	(31)	Multiple assay	DP	10.07 vs. 1.25 pg/mg (*p* < 0.001)
Vascular Endothelial Growth Factor A (VEGFA)	A potent pro-angiogenic mediator stimulated by pro-inflammatory stimuli and hypoxic condition.	(28)	Multiple assay	DF	14.09 vs. 17.18 pg/ml (*p* < 0.05)
Vasoactive intestinal peptide (VIP)	A potent anti-inflammatory factor produced by neurons and endocrine and immune cells, modulating both innate and adaptive immunity.	(46)	RIA	DP	67.12 vs. 77.96 pg/mg (*p* < 0.05)

RIA, Radioimmunoassay; MD, Microdialysis; IP, Irreversible pulpitis; RP, Reversible pulpitis; NP, Normal pulp; CE, asymptomatic caries exposure; DP, dental pulp tissues; GCF, gingival crevicular fluid; DF, dentinal fluid.
